# The ratio of cervical fluid and serum human chorionic gonadotropin as a predictor of abortion

**Published:** 2012-09

**Authors:** Shahrzad Zadehmodares, Nafiseh Baheiraei, Afsar Sharafi, Mehdi Hedayati, Mansoureh Mousavi

**Affiliations:** 1 *Infertility and Reproductive Health Research Center, Shahid Beheshti University of Medical Sciences (IRHRC), Tehran, Iran.*; 2 *Department of Tissue Engineering, School of Advanced Medical Technologies, Tehran University of Medical Sciences, Tehran, Iran.*; 3 *Cellular and Molecular Reasearch Center, Research Institute for Endocrine Sciences, Shahid Beheshti University of Medical Sciences, Tehran, Iran.*

**Keywords:** *Abortion*, *Cervical human chorionic gonadotropin*, *Cervical fluid*, *Prediction of abortion*, *Serum human chorionic gonadotropin*

## Abstract

**Background:** Progressions in science and technology have generated several methods for delaying preterm delivery and abortion; therefore, discovering an easy, non-invasive, practical, and non-expensive predictive factor can help us to perform preventive methods in healthy pregnant women, without any risk factors.

**Objective: **To indicate an appropriate index for predicting abortion in early pregnancy.

**Materials and Methods:** In a prospective study, 73 pregnant women who had a singleton pregnancy, had no complications or history of abortion or disease, and were referred to Mahdieh and Taleghani Hospitals between 2007-2009, were evaluated. Blood and cervical fluid samples were obtained thrice from all patients: at the first visit, after 1 week, and 1-2 weeks later. They were followed up until the 12^th^ week of gestation.

**Results:** Using the receiver-operator characteristic (ROC) curve analysis, 1.62 was obtained as the cut-off point for the cervical fluid: serum human chorionic gonadotropin concentration ratio; 14 patients (19.2%) experienced abortion, and 12 women (70.6%) had a ratio ≥1.62. Of the pregnant women with a ratio of <1.62, 3.6% had an abortion.

**Conclusion:** Pregnant women who do not show any signs of abortion and have a high cervical fluid: serum HCG concentration ratio are at risk of abortion; therefore, the cut-off point might be an appropriate index for predicting abortion in early pregnancy.

## Introduction

The overall incidence of abortion is about 10-15% of pregnancies (1, 2). Although many abortions are a result of fetal chromosomal disorders, a large number of abortions occur in the absence of any genetic abnormalities. Nevertheless, previous studies could not recommend a definite factor for predicting the probability of a spontaneous abortion (3). Progressions in science and technology have generated some available methods for delaying preterm delivery and abortion; therefore, discovering an easy, non-invasive, practical, and non-expensive predictive factor can help us perform the preventive methods in healthy pregnant women, without any risk factors.

Previous studies have evaluated various factors for predicting miscarriage, each introducing a different factor; pregnancy-associated plasma protein A (PAPP-A) (4, 5); macrophage inhibitory cytokine (6); progesterone (7, 8); and hyperglycosylated human chorionic gonadotropin (HCG) (9). 

However, their use is clinically limited because advanced technology is needed for performing methods based on these factors (10). Although measuring serum HCG is useful for indicating pregnancy and for predicting miscarriage, frequent blood sampling, which is necessary for predicting miscarriage, is not convenient for pregnant women. 

Therefore, finding a non-invasive, easy formula that does not require multiple blood sampling and is independent of knowing the exact time of the last menstrual period (LMP) would be beneficial. In a study by Takata *et al* a new concept was suggested for predicting miscarriage during early pregnancy, i.e., the cervical fluid: serum HCG concentration ratio (10). The present study aimed at verifying the predictive value of this formula as a predictor of early miscarriage.

## Materials and methods

In a prospective clinical study, 73 pregnant women who had a singleton pregnancy, had no complications or history of abortion or disease, and were referred to the prenatal clinics of Mahdieh and Taleghani Hospitals between October 2007 and April 2009 were evaluated during gestational weeks 4-12. This study was approved by the ethics committee of Infertility and Reproductive Health Research Center (IRHRC) at Shahid Beheshti University of Medical Sciences. A written consent was obtained from all patients after they were explained the aim and different steps of the study, and no extra expenditure was imposed on them. 

At the first visit, all patients underwent a routine pelvic examination and gave a blood sample for determination of the serum HCG concentration by using an enzyme-linked immunosorbent assay (ELISA) test. This sample was coagulated at room temperature, separated by centrifugation, and maintained at -30^o^C. Moreover, a cervical sample was obtained by washing the vagina and ectocervix with warm saline and rotating a cotton swab 2 times in the vaginal canal; the sample was diluted in 1 mL buffered-sulfate solution and centrifuged at a rate of 1000 rpm for 10 minutes. Supernatant was collected and stored at -30^o^C until the cervical fluid HCG concentration was determined using the ELISA test. The second blood and cervical samples were collected after 1 week, the third 1-2 weeks later, and if necessary, at the following visits. 

The levels of serum and cervical HCG were determined with a commercial enzyme immunoassay kit (HCG kit, Diagnostic Biochem Canada (dbc) company, Ontario, Canada; CV=5.2%, sensitivity: 0.7 IU/l). Gestational age was determined based on the LMP and transvaginal sonography during the first trimester. All patients were followed up every 3 weeks, and they received phone consultation. If they developed symptoms such as pain, bleeding, or spotting, or if any signs of abortion were observed, such as cervical dilatation and effacement confirming the diagnosis of unpreventable abortion samples, they were excluded from the study. 

If the patients were absent during the definite visit time, they were followed up by a phone call, and if they did not wish to continue being part of the study, they were excluded. In addition, during the study, if the patients developed any other complications of pregnancy, they were excluded from the study.

**Statistical analysis**

Data analysis was performed using the SPSS software version 15.00 for Windows, and an independent sample t-test was used for comparison of the 2 groups.

## Results

**Figure 1:**
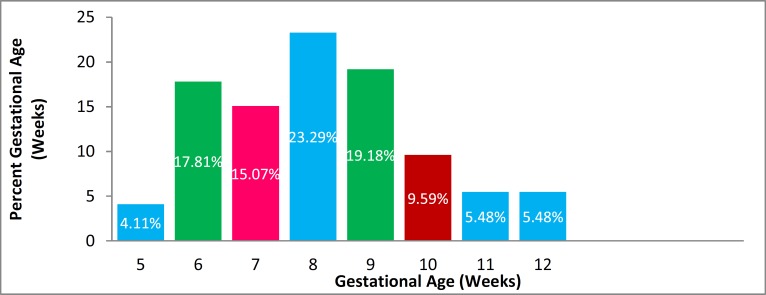
Distribution of gestational age and its percentage

Seventy-three pregnant women who were at 4-12 weeks’ gestation and were referred to our center were evaluated. The mean gestational age was 8 weeks (w) (8.14 w, SD=1.79); the distribution is shown in Figure 1. The mean gestational age in the group with normal pregnancy was 8.3 w (SD=1.7), and in the group with abortion was 7.4 w (SD=1.7), which was not significantly different (p=0.1). However, an association was observed between the gestational age and serum HCG concentrations (r=0.3, p=0.009).

Table I shows the mean cervical fluid HCG concentration, serum HCG concentration, and cervical fluid: serum HCG concentration ratio in the 2 groups (both normal and abortion groups). In the normal group, serum HCG concentration increased rapidly during gestational weeks 4-6 and peaked at gestational weeks 7-10, then decreased gradually and came to a plateau level. In this group, cervical fluid HCG concentration was slightly increased by progression in the pregnancy, which was not statistically significant (r=0.128, p=0.280). 

**Figure 2:**
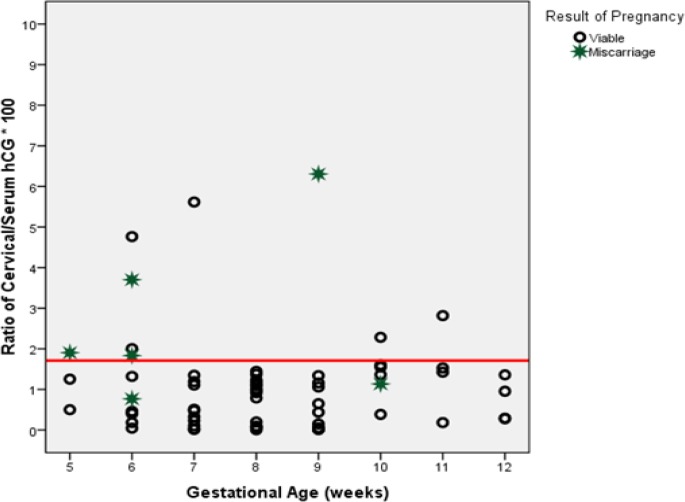
Distribution of the ratios of cervical fluid to serum human chorionic gonadotropin (HCG) concentrations in normal (viable) and miscarriage groups at 5-12 weeks’ gestation

Figure 2 shows the distribution of the cervical fluid: serum HCG concentration in both groups during gestation weeks 4-12. The cut-off point for the cervical fluid: serum HCG concentration ratio (1.62) was based on ROC curve analysis. In 56 cases (76.7%), the ratio was <1.62, and in 17 (23.3%), it was ≥1.62; at the end of the 12^th^ gestational week, 59 women (80.8%) had a normal pregnancy, and abortion occurred in 14 cases (19.2%). 

Of those who had an abortion, 12 of 14 patients (70.6%) had a ratio ≥1.62, whereas only 3.6% of women having a ratio <1.62 experienced abortion. Sensitivity, specificity, positive predictive value (PPV), negative predictive value (NPV), and accuracy of the cervical fluid: serum HCG concentration ratio as a predictor of abortion are indicated in table II. The mean age of the pregnant women was 27.22 years in the normal pregnancy group (SD=4.77), compared with 27.93 years in the abortion group (SD=5.6), which was not significantly different (p=0.631).

**Table I:** The comparisons of mean HCG concentration in cervical fluid and serum and its ratio based on the pregnancy groups

			**Amount**	**Mean**	**SD**	**p-value**
Cervical fluid HCG concentration (UL/ml)						
	Normal pregnancy		59	473.58	417.193	0.582
	Abortion		14	542.07	414.732	
Serum HCG concentration (UL/ml)						
	Normal pregnancy		59	61703.31	29033.21	0.001
	Abortion		14	13486	14050.38	
Cervical fluid/serum HCG concentration ratio						
	Normal pregnancy		59	0.921558	1.0369	0.002
	Abortion		14	6.321	5.268	

**Table II:** Sensitivity, specificity, positive predict value and negative predict value, Accuracy of cervical fluid: serum HCG in predicting early abortion

**Index**	**percentage**	**95% confidence interval (CI)**
Sensitivity	85.7%	56.2-97.5%
Specificity	91.5%	80.6-96.8%
PPV	70.6%	44-88.6%
NPV	96.4%	86.6-99.4%
Accuracy	90.41%	

## Conclusion

The present study is the second study on the accuracy of the cervical fluid: serum HCG concentration ratio in predicting the probability of miscarriage in early pregnancy. We analyzed the relationship between cervical and serum HCG concentration. Similar to a study performed in Japan in September 2005 (10). In this study, serum HCG concentration increased during gestational weeks 4-6 and peaked during gestation weeks 7-10, then gradually decreased, and came to a plateau level. In addition, the cervical fluid HCG concentration showed similar changes. 

In a previous study by Takata *et al* (10), 76 pregnant women were evaluated; 77.63% had a normal pregnancy at the end of the first trimester, and 22.37% had an abortion. The cut-off point for the ratio in this study was 1, while 83.3% of pregnant women with a ratio ≥1 and 3.4% of women with a ratio <1 experienced abortion. Although the mean cervical fluid: serum HCG concentration ratio in our study (1.957, SD=3.234) was significantly different from the measured mean value in the Japanese study (0.38, SD=0.461), the resulting cut-off points in both studies were similar-1 in the previous study compared to 1.62 in the present study. 

Although the greater SD values in our study showed more distribution in the study population, the sensitivity, specificity, PPV, and NPV were close to the findings of the previous study (88.2%, 94.9%, 83.3%, and 96.6%, respectively). According to the results of the present and previous studies (10), it should be emphasized that serum HCG concentration decreased in patients having clinical evidence of pregnancy loss; however, the increase in the cervical fluid: serum HCG concentration ratio in the first trimester detected before any symptoms of abortion suggest that asymptomatic pregnant women with a high concentration of HCG in cervical fluid and serum are at a risk of miscarriage.

Therefore, it could be an appropriate, practical, non-expensive, and available index for predicting the probability of early miscarriage. Use of this index can result in early diagnosis of abortion in the first trimester, giving physicians more time for determining the probable cause of abortion and for performing preventive methods such as hormonal support for better reassuring patients. It is suggested that further studies with larger sample sizes should be designed, even for patients experiencing recurrent abortions.
